# Immune Gene Expression Covaries with Gut Microbiome Composition in Stickleback

**DOI:** 10.1128/mBio.00145-21

**Published:** 2021-05-04

**Authors:** Lauren E. Fuess, Stijn den Haan, Fei Ling, Jesse N. Weber, Natalie C. Steinel, Daniel I. Bolnick

**Affiliations:** a University of Connecticut, Department of Ecology and Evolutionary Biology, Storrs, Connecticut, USA; b Texas State University, Department of Biology, San Marcos, Texas, USA; c Central European University, Department of Environmental Sciences and Policy, Budapest, Hungary; d College of Animal Science and Technology, Northwest A&F University, Yangling, People’s Republic of China; e University of Wisconsin–Madison, Department of Integrative Biology, Madison, Wisconsin, USA; f University of Massachusetts–Lowell, Department of Biological Sciences, Lowell, Massachusetts, USA; Duke University School of Medicine; University of Hawaii at Manoa

**Keywords:** animal microbiome, gut microbiome, host-microbe interactions, immune-microbiome interactions, immunity, microbial communities, microbiome

## Abstract

Commensal microbial communities have immense effects on their vertebrate hosts, contributing to a number of physiological functions, as well as host fitness. In particular, host immunity is strongly linked to microbiota composition through poorly understood bi-directional links. Gene expression may be a potential mediator of these links between microbial communities and host function. However, few studies have investigated connections between microbiota composition and expression of host immune genes in complex systems. Here, we leverage a large study of laboratory-raised fish from the species Gasterosteus aculeatus (three-spined stickleback) to document correlations between gene expression and microbiome composition. First, we examined correlations between microbiome alpha diversity and gene expression. Our results demonstrate robust positive associations between microbial alpha diversity and expression of host immune genes. Next, we examined correlations between host gene expression and abundance of microbial taxa. We identified 15 microbial families that were highly correlated with host gene expression. These families were all tightly correlated with host expression of immune genes and processes, falling into one of three categories—those positively correlated, negatively correlated, and neutrally related to immune processes. Furthermore, we highlight several important immune processes that are commonly associated with the abundance of these taxa, including both macrophage and B cell functions. Further functional characterization of microbial taxa will help disentangle the mechanisms of the correlations described here. In sum, our study supports prevailing hypotheses of intimate links between host immunity and gut microbiome composition.

## INTRODUCTION

Diverse communities of commensal microbiota are associated with a range of vertebrate organ systems, such as the respiratory tract (1), skin (2), and digestive tract (3). Each of these distinct communities can contribute to host physiological function and fitness (e.g., 4, 5) but can cause pathology when disrupted (e.g., 6). Conversely, host function, including the host immune system, also has effects on the composition of the microbiota, maintaining mutualists to avoid dysbiosis while also eliminating disease-causing pathogens (7, 8). Because host immune and physiological functions often entail changes in gene expression, these reciprocal interactions between host function and microbiome structure may be revealed by examining host gene expression (9, 10). Preliminary studies suggest that changes in the microbiome can affect host gene expression (9) and vice versa (11, 12). Still, much of what is known regarding cross talk between host gene expression and microbiota composition has been learned using reduced or single-taxa microbial models in a few host species (13–15). A potentially valuable next step would be to examine transcriptome-wide associations with variation in the entire gut microbial community. However, such studies are lacking because until recently, transcriptomic analyses were too expensive to allow sufficient statistical power (16).

Preliminary evidence suggests that host immunity, in particular, is closely linked to microbiota composition, likely through complex feedback networks (8, 17). Vertebrates’ mutualist bacteria promote key immune tolerance and regulatory pathways in a variety of organ systems (8). These include innate immunity in skin (18, 19), response to influenza in the respiratory tract (20), and development of gut-associated lymphoid tissue (GALT) and regulatory T cells in the intestines (5). Removal of, or changes in, these microbes compromises host immunity (21) or can lead to auto-immune disorders (22, 23). Furthermore, studies have shown the importance of commensal bacteria in regulating and educating adaptive immunity (24, 25), as well as contributing to development and homeostasis of innate immune cells (26–28). Conversely, on the part of the host, numerous immunological processes function in regulating microbiota composition, including physical barriers (i.e., host-secreted mucus layers; (29, 30), host-produced antimicrobial compounds (31), recognition molecules (i.e., pattern recognition receptors [PRRs] [32] and associated signaling [33]) and effector responses (i.e., secretion of antibodies [34]). Despite considerable preliminary knowledge regarding cross talk between host immunity and microbiota composition, the mechanisms of this feedback, particularly in regard to the roles of host gene expression, are not well described.

Advances in transcriptomics (RNAseq), have allowed for improved understanding of host function, including immunity and immune response, in diverse systems (35). These advancements can allow for the expansion of work investigating bidirectional interactions between microbiota and host immunity (here, “microbe-immune feedbacks”) beyond existing laboratory model systems with simplified microbial compositions (36–41). Despite these technical advances, RNAseq has yet to be broadly applied to investigating microbe-immune feedbacks, particularly in complex contexts. A few studies have indicated correlation between microbiome composition and expression of immune genes (42, 43). One such study screened a diversity of microbial species for their effects on host gene expression (whole transcriptome using Affymetrix arrays), demonstrating complex immunomodulatory effects of symbiotic microbes (13). However, these studies mostly document the effects of simplified microbiota or even monocultures. Only one study has examined more complex interactions, demonstrating strong associations between gene expression and microbiome composition in colonic epithelial cells, though these associations were limited to the localized colonic environment (9). Indeed, most studies examining feedback between microbiome composition and host gene expression have focused on localized gene expression, particularly in the gut epithelial tissue (44, 45). Consequently, we know little about the system-wide effects of microbiota composition on expression in distant immune-relevant tissues, and vice versa. Finally, few of these studies have examined the effects of genetic and environmental variation among hosts on these relationships.

Here, we report evidence for covariation between hosts’ gene expression and their gut microbiota, from a large sample of laboratory-bred three-spined stickleback (Gasterosteus aculeatus), a small fish native to northern temperate coastal marine and freshwater habitats. Like many vertebrates, individual stickleback harbor hundreds of microbial taxa (operational taxonomic units [OTUs]) in their intestines (46–49). The composition of this microbiota differs dramatically between cooccurring individuals within a given natural population and between populations (between neighboring lakes, adjacent lakes and streams, or marine versus freshwater [(46–48, 50–52]). For example, in one survey of a single natural population of stickleback, proteobacteria ranged from less than 5% to over 95% of the microbial community, depending on the individual host. This dramatic among-individual variation is associated with variation in diet, sex, genotype (at major histocompatibility complex [MHC] and other autosomal loci), helminth infection status, and interactions between these factors (46–49). Similar among-individual variation is observed within laboratory stocks of stickleback, whose microbiota is partly but not fully overlapping with the taxa seen in wild populations (48).

Here, we seek to test whether this among-individual variation in gut microbiota composition in the laboratory is associated with individuals’ immune gene expression. Using transcriptomic data generated from head kidneys (primary immune organ), we document correlations between immune gene expression and both microbiome diversity and proportion of key microbial families. Our results are some of the first to describe links between broad host immune functioning and microbiome structure in a nonmammalian vertebrate.

## RESULTS

### Correlations between gene expression and microbiome diversity.

**FIG 1:**
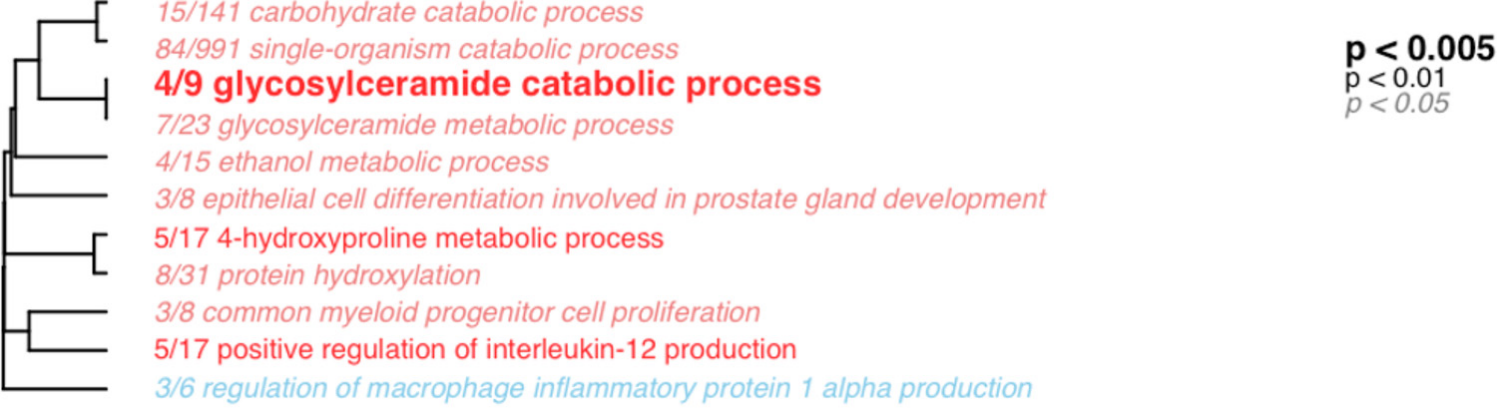
Hierarchical clustering of significantly enriched biological process gene ontology terms associated with genes significantly correlated with microbial diversity. Terms in red are positively enriched, and terms in blue are negatively enriched. Font style indicates level of significance.

Correlative analyses revealed strong associations between microbiota diversity and host gene expression, including expression of putative immune genes. Alpha diversity of the gut-associated microbiota was significantly correlated with 1,929 transcripts involved in a range of functions (Data set S1; ∼7.5% of all transcripts). These correlations were robust to experimental covariates; path analysis revealed that 1,014 (52.5%) of these correlations remained significant when accounting for sex, infection, mass, etc. We will henceforth discuss all 1,929 of the identified correlated transcripts. Of this total, 834 transcripts were positively correlated with microbial diversity and 1,095 were negatively correlated with diversity. Many of the correlated transcripts were involved in different arms of immunity (Table 1). Genes significantly correlated with microbiome diversity were significantly enriched for 11 biological process gene ontology (GO) terms, 10 positively (i.e., overrepresented processes which are increasing as a result of increased diversity of the microbiota based on tau values) and 1 negatively (i.e., overrepresented processes whose expression is lower in fish with high microbial diversity; Fig. 1). This included two terms involved in immunity that were positively correlated with diversity: “positive regulation of interleukin-12 production” and “common myeloid progenitor cell proliferation.” Genes that significantly contributed to enrichment of these two terms included receptor-type tyrosine-protein kinase FLT3 (ENSGACT00000004059), Toll-like receptor 9 (ENSGACT00000013443), tumor necrosis factor receptor superfamily member 5 (ENSGACT00000014780), interferon regulatory factor 8 (ENSGACT00000021099), and peregrin (ENSGACT00000001616). In contrast, the immune-related term “regulation of macrophage inflammatory protein 1 alpha production” and associated genes were expressed at lower levels in fish with more diverse gut microbiota. Significant genes included in this term were pyrin (ENSGACT00000027215), high mobility group protein B1 (ENSGACT00000027215), and transient receptor potential cation channel subfamily V member 4 (ENSGACT00000012089). In sum, microbial diversity was positively correlated with expression of genes associated with development of immune cells and regulation of interleukin-12 (IL-12) but negatively correlated with expression of genes associated with inflammatory processes.

**TABLE 1:** Examples of immune genes that were significantly correlated with diversity of gut-associated microbiota^*a*^

Gene	Ensembl ID	Immune function	tau value	*P* value
C-C motif chemokine 4	ENSGACT00000000554	Chemoattractant (NK cells, monocytes, etc.)	0.197	4.76e-07
Interferon regulatory factor 4	ENSGACT00000021099	Transcriptional activator (antiviral)	0.152	9.96e-05
Complement C3	ENSGACT00000026259	Complement cascade (innate immunity)	0.133	7.65e-04
Eosinophil peroxidase	ENSGACT00000022724	Antibacterial activity	–0.175	7.68e-06
Interleukin-11	ENSGACT00000013923	Hematopoietic stem cell proliferation	–0.152	0.00134
NLR family CARD domain-containing protein 3	ENSGACT00000001559	Negatively regulates innate immunity	–0.143	0.00272

a A full list of significantly correlated genes can be found in Data set S2.

10.1128/mBio.00145-21.4 DATA SET S1 List of transcripts which were significantly correlated with alpha diversity of the gut microbiome. Transcript ID, annotation data, *P* value, and tau values are listed. Download Data Set S1, XLSX file, 0.1 MB. Copyright © 2021 Fuess et al. 2021 Fuess et al. https://creativecommons.org/licenses/by/4.0/ This content is distributed under the terms of the Creative Commons Attribution 4.0 International license.

10.1128/mBio.00145-21.5 DATA SET S2 List of transcripts which were significantly correlated with each of the top 15 most correlated microbial families. Transcript ID, annotation data, *P* value, and tau values are listed. Download Data Set S2, XLSX file, 0.4 MB. Copyright © 2021 Fuess et al. 2021 Fuess et al. https://creativecommons.org/licenses/by/4.0/ This content is distributed under the terms of the Creative Commons Attribution 4.0 International license.

**FIG 2:**
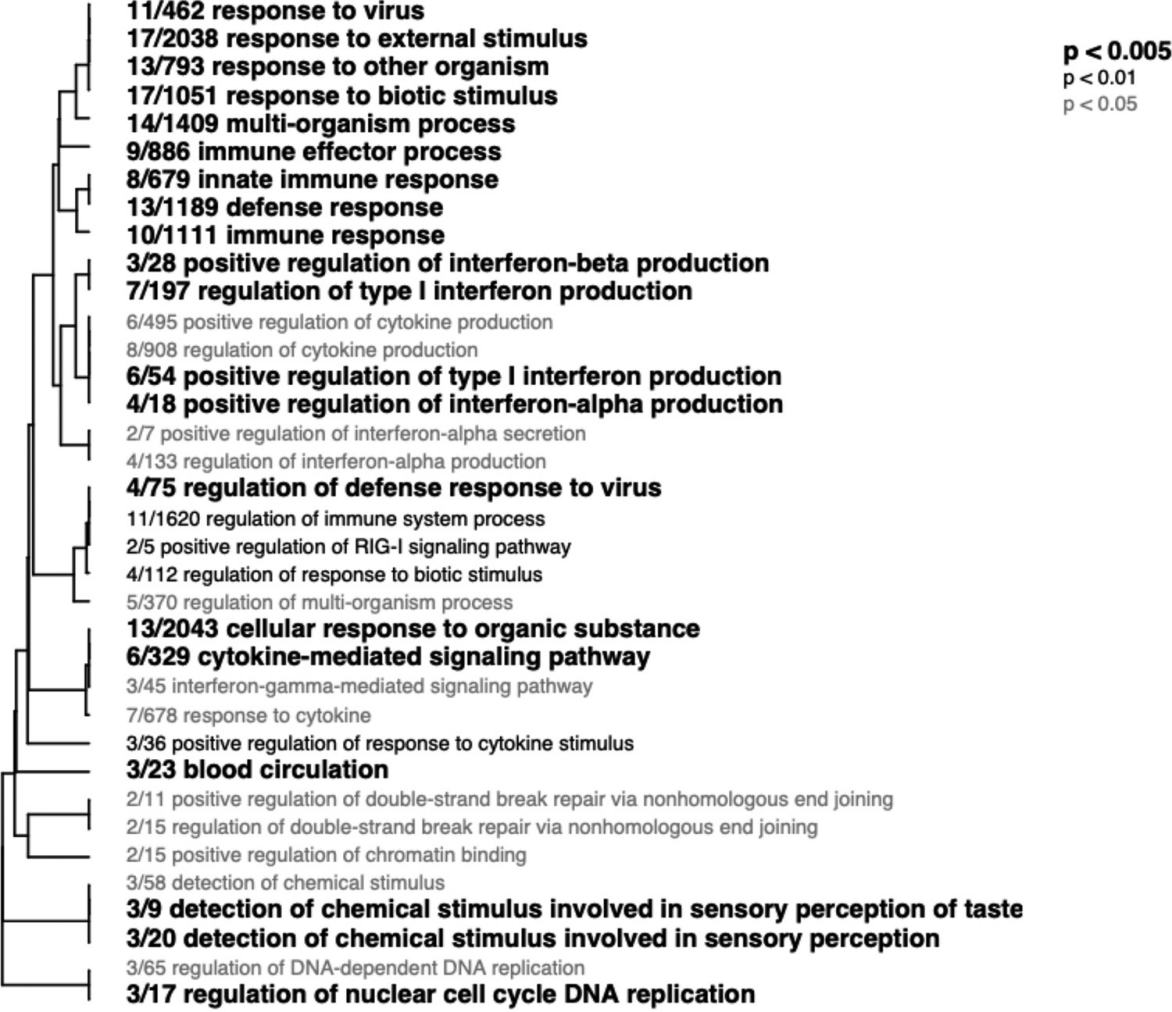
Hierarchical clustering of significantly enriched biological process gene ontology terms associated with genes in module 7 (positively associated with microbial diversity). Font style indicates level of significance.

Coexpression analyses revealed strong associations between microbiota diversity and host gene expression, and immune genes in particular. The resulting network comprised 10 modules plus a “gray” module, module 11, containing transcripts that did not fit into any existing modules. These modules ranged in size from 44 to 18,227 transcripts. The largest of these modules (module 10) likely represents groups of housekeeping genes with low variable expression. Two modules were significantly positively correlated with microbial diversity, module 2 (*r* = 0.11, *P* = 0.03) and module 7 (*r* = 0.12, *P* = 0.02; Fig. S2). Module 2 (1,344 transcripts) was significantly enriched for 76 biological process gene ontology terms involved in a wide diversity of processes, indicating its roles in basic cellular homeostasis. Some of the largest groups of these terms included those involved in translational initiation, cytosolic transport, cellular component biogenesis, and electron transport chain (Fig. S3). The biological meaning of this module is ambiguous. In contrast, the much smaller module, module 7 (48 transcripts), was enriched for 36 biological process gene ontology terms, 20 of which were related to immunity and defense (Fig. 2). Thus, we concluded that module 7 consists of coregulated genes predominately involved in immune function. Enriched terms included those involved in interferon production (“positive regulation of type-I interferon production,” “positive regulation of interferon alpha production,” interferon gamma-mediated signaling pathway,” etc.) and cytokine signaling (“cytokine-mediated signaling pathway,” “regulation of cytokine production,” etc.), as well as other general immune GO terms (“immune response,” “immune effector process,” “innate immune response,” etc.). Thus, fish with more diverse microbiota generally exhibited higher coexpression of these categories of immune genes. Consequently, coexpression analyses indicate strong positive association between host gene expression and a diverse network of genes involved in immunity, with emphasis on interferon and cytokine signaling.

10.1128/mBio.00145-21.2 FIG S2 Tombstone plot displaying correlations between coexpression network modules and alpha diversity or microbial family diversity (for families of interest). Only significant values are displayed. Download FIG S2, PDF file, 0.1 MB. Copyright © 2021 Fuess et al. 2021 Fuess et al. https://creativecommons.org/licenses/by/4.0/ This content is distributed under the terms of the Creative Commons Attribution 4.0 International license.

10.1128/mBio.00145-21.3 FIG S3 Hierarchical clustering of significantly enriched biological process gene ontology terms associated with genes in module 2 (positively associated with microbial diversity). Font style indicates level of significance. Download FIG S3, PDF file, 0.04 MB. Copyright © 2021 Fuess et al. 2021 Fuess et al. https://creativecommons.org/licenses/by/4.0/ This content is distributed under the terms of the Creative Commons Attribution 4.0 International license.

### Correlations between gene expression and relative abundance of specific taxa.

**FIG 3:**
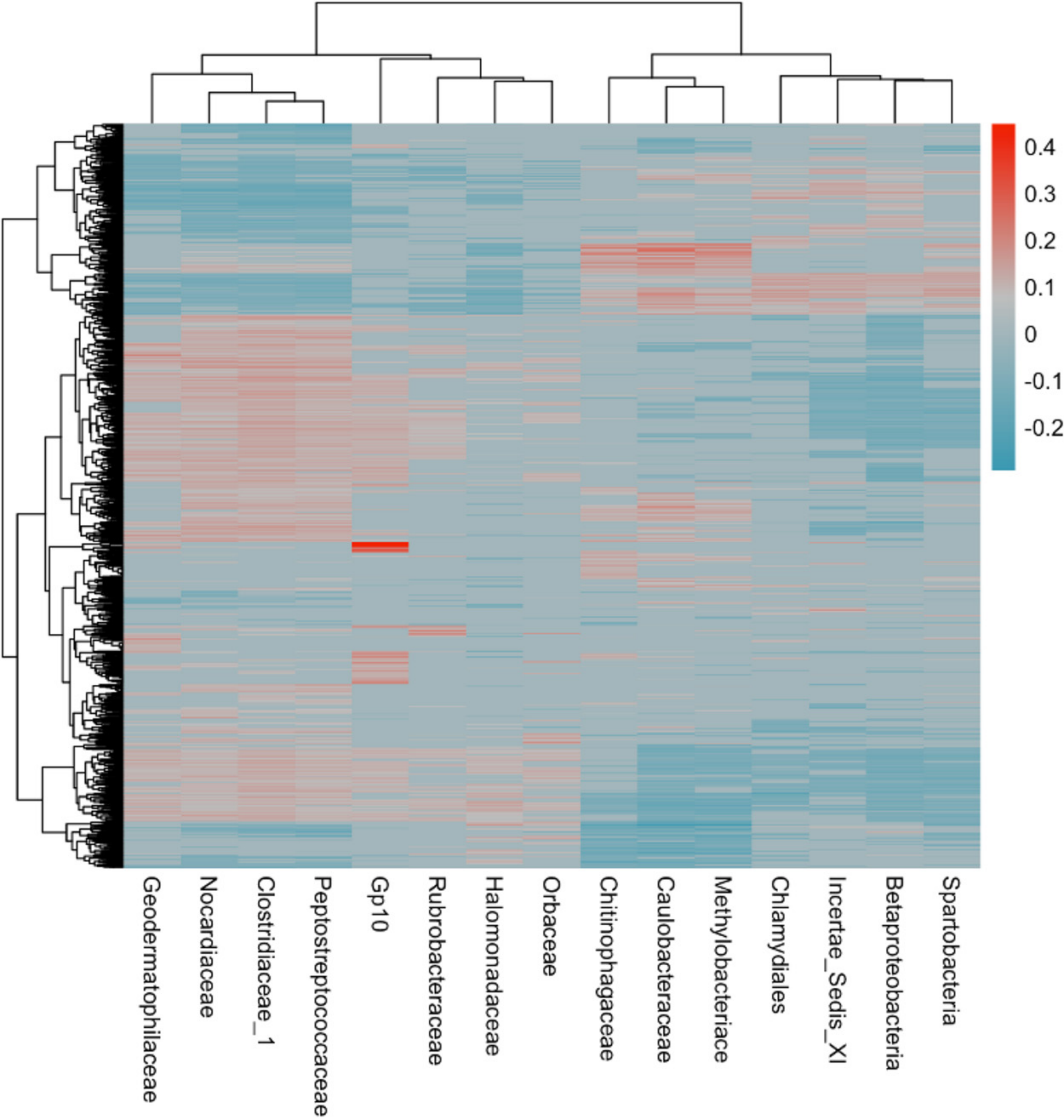
Heat map of correlations between families and genes of interest. Values shown are Kendall’s tau for each correlation. Gray fill indicates nonsignificant correlations. Rows and columns are hierarchically clustered using default parameters.

Initial analysis of correlations between microbial families and host gene expression identified 507,317 significant associations out of 7,893,582 possible pairwise correlations between the relative abundance of a given family and a specific gene (∼6.4% of total correlations run, slightly but very significantly more than the 5% expected from type II error alone). We took a conservative approach and further examined only correlations between approximately the top 5% (approximate) most correlated microbial families (*n* = 15) and genes (*n* = 1,297). Combined, these families were correlated with a total of 1,263 of the 1,297 top correlated genes (Fig. 3; Data set S2). Again, most of these filtered relationships were robust to covariates (Table 2); thus, here we will discuss all significant results from the initial analysis.

**TABLE 2:** Summary of statistically significant correlations between the top 5% most correlated families and genes and the number of correlations which remain significant following covariate analyses using structural equation modeling (SEM analysis)

Microbial taxa	No. of significantly correlated genes	
	Correlation analysis	SEM analysis
*Betaproteobacteria* (unclassified)	639	459
*Caulobacteraceae*	591	484
*Chitinophagaceae*	389	182
*Chlamydiales* (unclassified)	376	210
*Clostridiaceae* 1	926	662
*Geodermatophilaceae*	661	427
Gp10 (unclassified)	505	217
*Halomonadaceae*	393	324
*Incertae Sedis XI*	480	316
*Methylobacteriaceae*	523	410
*Nocardiaceae*	893	658
*Orbaceae*	306	174
*Peptostreptococcaceae*	908	594
*Rubrobacteraceae*	351	187
*Spartobacteria* (unclassified)	488	432

**FIG 4:**
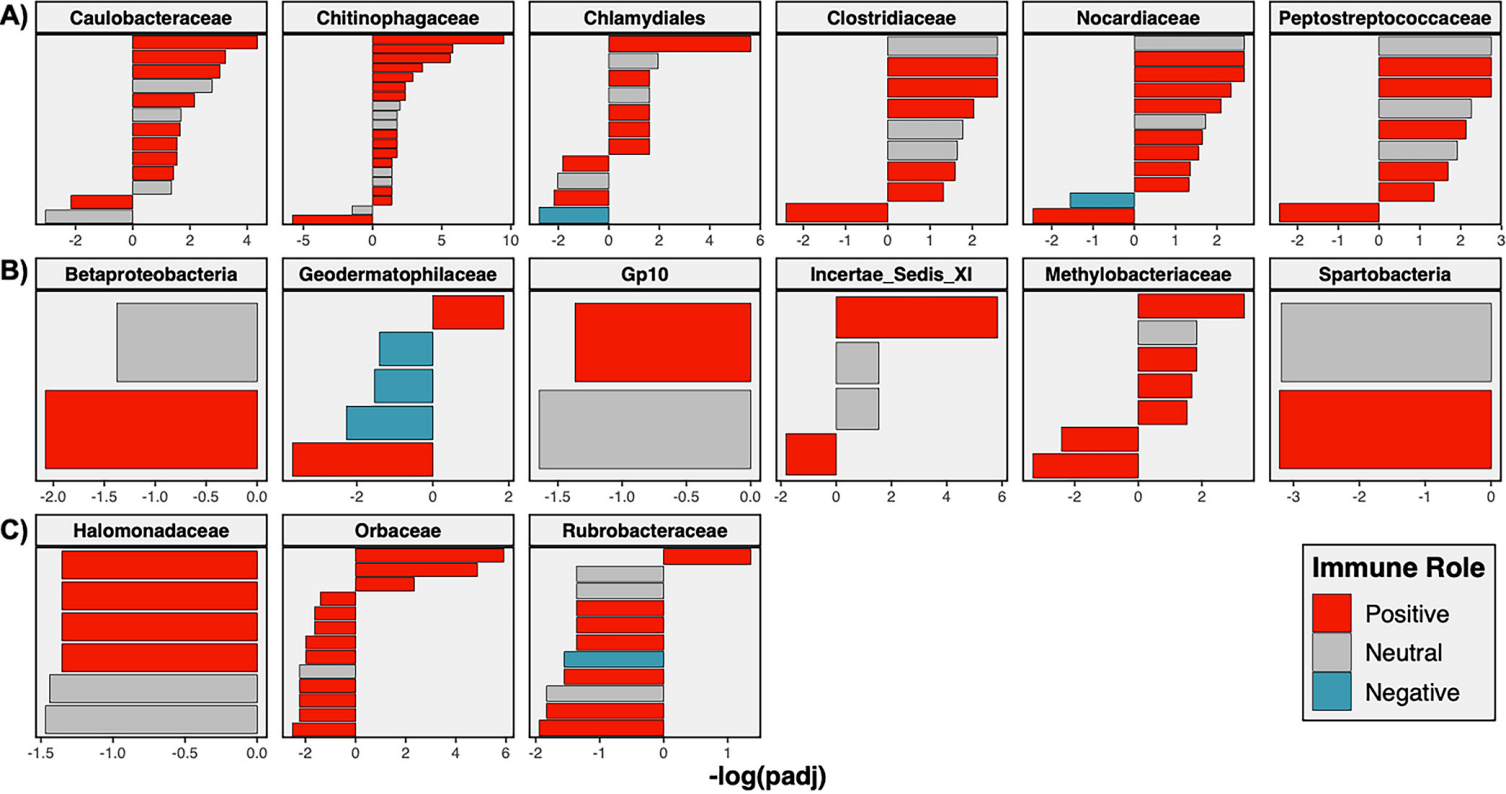
Significance and enrichment of immune-associated biological process GO terms associated with each of the 15 microbial families of interest. (A to C) Families are classified into the following three groups: (A) families negatively associated with immunity, (B) families neutrally associated with immunity, and (C) families positively associated with immunities. Each bar indicates a significantly enriched biological GO term; color indicates the association of the term with immunity (red indicates terms which positively contribute to immune functions, blue indicates negative terms, and gray indicates neutral); the direction of bar indicates positive or negative enrichment; the magnitude of bars indicates the negative log of the adjusted *P* value.

Gene ontology enrichment of genes correlated with each family revealed significant patterns of enrichment of immune processes. The 15 families fell into one of three categories based on gene ontology enrichment analyses—positively immune associated, neutrally immune associated, or negatively immune associated (Fig. 3; Data set S3). Families such as *Rubrobacteraceae*, *Orbaceae*, and *Halomonadaceae* showed significant negative associations with immunity. Genes correlated with *Rubrobacteraceae* and *Halomonadaceae* abundance were significantly negatively enriched for immune-associated biological process GO terms such as “lymphocyte aggregation,” “response to interleukin-15”, and “myeloid lymphocyte migration.” Similarly, *Orbaceae* abundance was significantly correlated with genes negatively enriched for immune terms, including “pro-B cell differentiation,” “positive regulation of interferon alpha secretion,” and “hummoral immune response.” In contrast, abundance of six microbial taxa, including *Caulobacteraceae* and *Chlamydiales*, was positively associated with immune processes. Genes correlated with these taxa were positively enriched for immune-associated biological process GO terms such as “positive regulation of B cell differentiation,” “positive regulation of macrophage activation,” “positive regulation of interleukin-12 production,” and “myeloid progenitor cell differentiation.” Six microbial families had mixed (i.e., neutral) associations with biological processes (Fig. 4). Based on enrichment analysis of the correlative results, abundance of microbial taxa can have complex effects on host gene expression, dependent on the taxon identity.

10.1128/mBio.00145-21.6 DATA SET S3 Full gene ontology enrichment results for correlations between transcripts and each of the top 15 most correlated microbial families. Download Data Set S3, XLSX file, 0.1 MB. Copyright © 2021 Fuess et al. 2021 Fuess et al. https://creativecommons.org/licenses/by/4.0/ This content is distributed under the terms of the Creative Commons Attribution 4.0 International license.

Nine of ten WCGNA coexpression modules were correlated with one or more of the 15 identified microbial families of interest (Fig. S2). Module 7, the immune functioning module significantly correlated with diversity, was only correlated with abundance of *Spartobacteria*. Of the nine modules that were correlated with one or more family of interest, most had broad biological functions. Exceptions to this included module 1, which was enriched primarily for biological processes involved in tetrapyrrole metabolism and inorganic anion transport. Module 1 was significantly positively correlated with *Betaproteobacteria* (*r *= 0.17, *P = *0.0009) and significantly negatively correlated with *Chitinophagaceae* (*r *= 0.11, *P = *0.03) and *Peptostreptococcaceae* (*r*=–0.12, *P = *0.01). Additionally, module 9, which was enriched for numerous terms involved in wound healing, defense, and immune response, was positively correlated with both *Chitinophagaceae* (*r *= 0.11, *P = *0.04) and *Orbaceae* (*r *= 0.11, *P = *0.03) Coexpression analyses suggest that microbial families are related to broad networks of host gene expression in complex, family-dependent patterns.

## DISCUSSION

### Microbiome diversity is positively associated with host immunity.

Numerous studies, mostly of laboratory mice, indicate that vertebrates’ microbiome composition is both a function of their immune genotype and phenotype (11, 53–55) and, in turn, can modify host immune development and response (55–57). Despite this, the mechanisms of these relationships, as well as patterns of microbe-immune feedback in more complex and diverse systems, are not known. Here, we provide some of the first evidence of the roles of host gene expression in mediating microbe-immune feedback in a nonmammalian system. Based on existing evidence, we expect correlations between gut microbiota composition and host immunity, as measured by gene expression in immunological tissues. This should be true even when examining tissues that are anatomically separated from the organ(s) containing the microbiota, because localized interactions in the gut may alter system-wide immune traits. Consistent with this expectation, we find that alpha diversity of *G. aculeatus* gut microbiota was significantly correlated with expression of a large number of host genes expressed in the head kidney, many of which had functions in immunity. Coexpression analysis further confirmed strong associations between a broad diversity of immune components and microbial diversity. Our results collectively confirm that, broadly speaking, host immunity (measured by transcriptomics) and microbiome diversity are positively associated. This finding is agreement with, and expands upon, past studies of *G. aculeatus*, which found that fish from some populations reared with conventional microbiota communities had higher neutrophil activity in their guts than germfree fish (51). However, our results are unique in highlighting the far-reaching effects of gut microbiome on host immune gene expression; gut microbiome composition was correlated with gene expression broadly, and expression of immune genes especially, in the anatomically distant head kidney (located cranially near the gills). Correlations between gut microbiota and gene expression in head kidney tissue indicate significant cross talk between immune cell development and microbiota composition, as the head kidney is primarily involved in the development of a range of immune cells (58). Consequently, our findings suggest complex effects of gut microbiome on the development of host immune cells and, consequently, host immune function.

Gene ontology analysis of significantly correlated genes emphasized the relationship between microbiome diversity and expression of specific immune genes in *G. aculeatus*. “Positive regulation of interleukin-12 production” was positively associated with microbiome diversity. This is in accordance with past studies demonstrating effects of pro- and prebiotic compounds on interleukin-12 production (59–61). Furthermore, “common myeloid cell progenitor differentiation” was positively associated with microbial diversity. Past studies have demonstrated strong effects of the microbiota on myeloid cell development. Commensal bacteria increase the amount and division of myeloid progenitor cells (62); increased myelopoiesis (62) and bone marrow myeloid cell abundance (27) are positively associated with microbiome complexity. Our transcriptomic results indicate similar relationships between gut microbiota and development of immune cells in a major teleost hematopoetic organ, the head kidney. Finally, “regulation of macrophage inflammatory protein 1-α (MIP1-α)” was negatively associated with microbiome diversity. MIP1-α is an inflammatory chemokine (63). Inflammation is well known to be linked with dysbiosis of the microbiome (7); reduced microbial diversity is associated with many inflammatory conditions of the gut (7, 64). In sum, our analyses revealed significant connections between microbiome diversity and host immunity as measured by transcriptomics, though the mechanism and direction of causation of these relationships requires further study.

### Microbial taxa have opposing effects on host immune gene expression.

In addition to highlighting significant microbe-immune feedback associated with diversity of the gut microbiota, our study also provides substantial initial evidence of the effects of specific microbial taxa on host gene expression and immunity. Abundance of specific microbial taxa was correlated with a wide array of host genes, with functions in a diversity of biological processes. Indeed, coexpression analysis demonstrated strong associations between abundance of particular microbial families and modules involved in broad host functioning. The exact nature of these associations varied among microbial families, with certain groups of microbial families displaying opposing trends of correlations to both individual genes, and coexpression modules. Specific microbial taxa are known to effect broad host functioning (5, 65–67). Our results are among the first to highlight the complex nature of specific host-microbe interactions, the importance of host gene expression in mediating these interactions, and the effects of these relationships on a diversity of functions. Here, we will specifically focus on variation in association between microbial taxa and expression of genes involved in host immunity.

Gene ontology analysis of associations between families and genes of interest revealed clear patterns of associations between microbial family abundance and host immune gene expression. Microbial taxa fell into three groups—those positively correlated, negatively correlated, or neutrally associated with immune gene expression. It is known that certain groups of commensal bacteria, such as segmented filamentous bacteria (68), are positively associated with aspects of host immunity, while others, including Bacteroides fragilis (69), induce protective tolerogenic responses, suppressing immunity (13). Furthermore, pathogenic bacteria may suppress (70, 71) or induce (72, 73) host immunity during infection. These relationships are often highly context dependent, and the mechanisms of these relationships are poorly understood. Here, we break down observed relationships between specific microbial family abundance and host immunity, highlighting the need for increased functional understanding of these taxa in order to improve mechanistic knowledge of the microbiome and its effect on host function.

Abundance of six microbial taxa was significantly positively associated with expression of immune genes in our study. These families were *Caulobacteraceae*, *Chitinophagaceae*, *Chlamydiales*, *Clostridiaceae*, *Nocardiaceae*, and *Peptostreptococcaceae*. Half of these families (*Caulobacteraceae*, *Chlamydiales*, and *Nocardiaceae*) are well described for their association with disease and other pathologies (74–76). Specifically, the family *Nocardiaceae* is known to contain some opportunistic pathogens (77), including microbes that induce nocardosis in fish (78). Positive associations between these taxa and expression of host immune genes may be indicative of the pathogenic nature of these microbes, which would induce host immune responses.

The remaining three families that were positively associated with host immunity, *Chitinophagaceae*, *Clostridiaceae*, and *Peptostreptococcaceae*, have diverse roles in microbial communities. Members of the family *Chitinophagaceae* are often described as components of the commensal microbiota of aquatic species, including lampreys (79), and aquatic amphibians (80), though its function is poorly understood. Studies have highlighted beneficial functions of these microbes in degrading chitin (81), which may explain their ability to inhibit growth of the common fungal amphibian pathogen Batrachochytrium dendrobatidis (80). Members of the family *Clostridiaceae*, specifically, segmented filamentous bacteria (SFB), have well-documented effects on mammalian immunity. SFB are capable of enhancing Th17 cell responses (68) and promoting increased IgA production in mice (82). Finally, *Peptostreptococcaceae* is a poorly described yet diverse microbial taxon which is often associated with the vertebrate gut microbiome. Much of what is known regarding this family is based upon extensive research regarding a single representative species, Clostridium difficile (83). However, this family is immensely diverse (84), necessitating further functional study to understand broader associations of members of this taxon with host immunity. Indeed, further functional classification and controlled mechanistic studies will prove fruitful in understanding positive associations between the taxa identified here and host immune functioning.

In contrast to those taxa identified as positively associated with host immune gene expression, three families could be classified as negatively associated with host immunity—*Halomonadaceae*, *Orbaceae*, and *Rubrobacteraceae*. All three of these microbial families are poorly described, and two (*Halomonadaceae* and *Rubrobacteraceae*) have been primarily described as environmental microbes. Members of the family *Halomonadaceae* are well described as halophiles (85). Although some preliminary studies indicate a potential pathogenic role of members of this group (86–88), the roles of this family in microbiome composition are not well understood. Both *Rubrobacteraceae* and *Orbaceae* have been documented as members of insect gut microbiomes, found in termites (89), and bees (90), respectively. *Orbaceae* in particular has been described to have negative impacts on bee colony productivity (91, 92). Our study is the first, to our knowledge, to report the presence of these microbes in the vertebrate gut microbiome. Further functional characterization of these three taxa is necessary to interpret their associations with host immunity in the *G. aculeatus* system.

Finally, it is worth noting common trends in correlations between expression of genes involved in specific immune components and microbial family abundance. Genes associated with several immune components were correlated with at least a third of the significant microbial families, as revealed by gene ontology analysis. Many of these components have also been linked to microbiome function or composition. Genes involved in myeloid progenitor cell differentiation, regulation of interleukin-12 secretion/production, interferon gamma production, pro-B cell differentiation, and positive regulation of macrophage activation were commonly correlated with microbial family abundance. We have previously discussed the importance of both IL-12 and myeloid progenitor cells in the maintenance of gut-microbiome composition. The production of interferon gamma (IFN-γ) has been both positively and negatively linked to various microbial components; IFN-γ production decreased in piglets treated with a probiotic bacterium (93) and immunomodulary compounds from other bacteria (94). In contrast, IFN-γ^+^ CD8 T cells are induced by commensal bacteria in human guts (95).

We observed frequent correlation between microbial family abundance and expression of immune genes associated with B cell processes and macrophage activity. Microbiota are known to have profound effects on B cell processes, including diversification, production of IgA, and differentiation of regulatory B cells (24, 96). Furthermore, on the part of the host, B cell production of IgA in particular is essential for maintenance of gut microbiome composition by restricting commensal growth and maintaining a diverse composition (97). Similarly, in some teleost fish such as rainbow trout, IgT is known to play important roles in microbiome homeostasis (98). Similar bi-directional relationships are known to exist between macrophages and gut microbiota. Microbial metabolites such as butyrate can modulate and reduce macrophage activity to promote tolerance of commensal bacteria (56). Macrophages can also shape gut microbiota structure, potentially by discriminating between commensal and pathogenic microbes (99). In sum, the most striking patterns of correlation between immune gene expression and microbial family abundance add to the existing literature supporting the roles of these specific arms of immunity, and specific cell types, in microbiome maintenance.

**Conclusions.** Here, we document one of the first investigations of correlations between natural gut microbiome composition and host transcriptomic gene expression in a nonmammalian vertebrate. Our results detail extensive correlation between the host’s transcriptome and both diversity and proportion of specific microbial families. Notably, these associations exist despite spatial separation between the microbiota and the organ where we measure expression, highlighting the systemic changes induced by gut microbiota. Both diversity and microbial family proportion are strongly correlated with expression of a diversity of host immune genes. Associations between immunity and microbial diversity likely reflect both the effects of a healthy, diverse microbiome on the host immune system and the need for a robust immune system to maintain this diversity. Trends in correlation between abundance of specific microbial families and host immune gene expression identified groups both positively and negatively associated with host immunity. Many of these trends support previous studies from other systems. However, increased functional understanding of microbial taxa is needed to interpret these trends. In sum, our results highlight the immense interconnectivity between host gene expression and gut microbiome composition, specifically in regard to immune function. These results also highlight the utility of the transcriptomic tools in enabling studies of microbe-immune feedbacks in wild populations and nonmodel animals.

### Data availability.

Raw data and code for all analyses described in the manuscript can be found on GitHub (https://github.com/lfuess/MicrobiomeMS).

## MATERIALS AND METHODS

### Experimental design.

Full details of data collection can be found in Ling et al. (49) and Fuess et al. (100). Briefly, we collected reproductively mature fish from two lakes on Vancouver Island, British Columbia, Canada (Roberts and Gosling Lakes). Eggs were removed from gravid females and fertilized using sperm from testes collected from males from the same lake (pure F1s) or from the other lake (F1 hybrids). Eggs were shipped back to the University of Texas at Austin, hatched, and reared to reproductive maturity. The resulting adults were again artificially crossed to generate F2 hybrids consisting of intercrosses (F1×F1 hybrids) or backcrosses (ROB×F1 or GOS×F1). The resulting generation was reared for 1 year and then experimentally exposed to Schistocephalus solidus cestodes, following standard procedures (101, 102). Then, 42 days postexposure, fish were euthanized and data were collected for a number of phenotypic metrics, including sex, mass, and infection status/load. We also dissected head kidneys for immune transcriptomic analysis. In fish, the head kidney, or pronephros, is a primary immune organ functioning primarily as a lympho-myeloid compartment (58). As is indicated by the name, this structure is located in the cranial region of the fish, near the gills, separating it considerably from the gut. Guts were dissected using sterile protocols for microbiome composition analysis (49).

### Transcriptomic analysis.

RNA was extracted from one head kidney, and sequencing libraries were generated following methods described in Fuess et al. (100). We extracted RNA from this organ using the Ambion MagMAX-96 total RNA isolation kit following a modified version of the manufacturer’s protocol. A DNA removal step was preformed using TURBO DNase. RNA yield was quantified using a Tecan NanoQuant Plate. TagSeq RNA sequencing libraries were constructed using a modified version of methods described in Lohman et al. (103), detailed in Fuess et al. (100). Libraries were sequenced on a HiSeq 2500 instrument at the Genomics Sequencing and Analysis Facility of the University of Texas at Austin, Texas.

Resulting sequencing reads were processed using the iRNAseq pipeline (104). Reads were aligned to version 95 of the stickleback genome on Ensembl using Bowtie 2 software (105), and any samples with less than 500,000 aligned reads were discarded (final *n* = 393). A matrix of normalized read counts was generated using the R package DESeq2 (106). This normalized read count matrix was used for all subsequent analyses (correlations, path analyses, and WGCNA). Information about the resulting read counts per individual, annotation, and other metrics of transcriptome information are reported in Fuess et al. (100).

### Gut microbiota analyses.

Full details regarding sampling and analysis of gut microbiota composition can be found in Ling et al. (49). To summarize, DNA was extracted from the entirety of collected stickleback intestines (*n *= 693 fish) using MoBio Powersoil DNA isolation kits. From these data, 16S rRNA amplicons were generated for the V4 hypervariable regions. Sequencing was performed on an Illumina Miseq platform at the Genomic Sequencing and Analysis Facility at the University of Texas at Austin, Texas. The resulting reads were processed using standard procedures in the mothur software package (v.1.39.1) (107). OTUs were identified using the UCLUST algorithm based on 97% similarity. The relative proportion of microbial taxa (calculated at the level of family) was calculated as the proportion of total OTU reads from a sample representing a given family compared to the total number of OTUs for a sample. Data were rarefied to 2,000 sequences, and Chao1 alpha diversity was calculated using the R package phyloseq (108). Information about the resulting number of microbial OTUs, counts, and read depth per individual are reported in greater detail in Ling et al. (49), who examined the microbiota’s response to cestode infection and host genotype.

### Correlative analyses.

We tested for correlations between microbiome composition and host gene expression. All statistics were conducted in R (v.3.6.1). First, we correlated the gene expression of all expressed genes to alpha diversity using a Kendall’s rank correlation. Genes with *P* values less than 0.05 were considered significant for further analyses. Next, to identify families of microbes that are highly associated with gene expression, we correlated the gene expression of all expressed genes to the relative proportion of each microbial family, again using a Kendall’s rank correlation. This resulted in thousands of significant associations between families and genes, many of which may be false positives due to the exceptionally large number of tests run (considering the combinations of many transcripts against many microbes). We concluded that the most conservative approach would be to select the top 5% (approximate; ties accounted for) most significantly correlated families (*n* = 15) and genes (*n* = 1,290) and consider only relationships between these two groups for further analyses.

To assess the effects of covarying factors (i.e., successful cestode infection, sex, host genotype [cross]) and ensure that correlations were not the result of spurious covariate effects, we also conducted a path analysis using the R package sem (109). Potential covariates which may have confounded relationships detected by the correlative analyses were included in the model—sex, infection, mass (log-transformed), and cross-direction. The full model structure can be found in the supplemental material (Fig. S1).

10.1128/mBio.00145-21.1 FIG S1 Schematic of the structural equation model that was used to assess robustness of covariance between gene expression and microbial diversity or taxon abundance. Download FIG S1, PDF file, 0.03 MB. Copyright © 2021 Fuess et al. 2021 Fuess et al. https://creativecommons.org/licenses/by/4.0/ This content is distributed under the terms of the Creative Commons Attribution 4.0 International license.

### Gene ontology analyses.

To determine the biological processes most correlated with microbiome diversity and composition, we conducted gene ontology analyses. We assessed enrichment of biological process GO terms, using the R script GO-MWU (110). To identify biological processes enriched as a result of variation in microbiome diversity, we conducted gene ontology enrichment analyses using the tau values for all significantly correlated transcripts; all other genes were assigned a value of 0. We used a similar approach for assessing biological process terms enriched in relation to relative family proportion for each of our families of interest. Gene ontology analyses were conducted using GO terms associated with stickleback gene annotations (see reference 53) and performed independently for each family. Input for this analysis was a matrix comprised of tau values for all significant correlations between a given family and the top 5% of genes (all other genes assigned a value of 0).

### Coexpression analyses.

We used coexpression analyses to identify groups of coexpressed host genes that were significantly correlated with microbiome diversity or relative proportion of microbial families (using only the 15 most significantly correlated families identified previously). Coexpression analyses were run using the R package WGCNA (111). We constructed a signed network using bicor analyses and the following parameters: soft power = 12; minimum module size = 30; deepSplit = 2; dissimilarity threshold = 0.2. The resulting network was correlated with microbiome diversity and relative family proportion using a bicor correlation. Modules with significant correlations (*P < *0.05) were analyzed for enrichment of biological processes using gene ontology enrichment (GO-MWU; default parameters for analysis of WGCNA modules).
