# Supplementary figures and images for: Immune Gene Expression Covaries with Gut Microbiome Composition in Stickleback
Source: mBio. 2021 May 4;12(3):e00145-21. doi: 10.1128/mBio.00145-21 (PMC8262870; doi:10.1128/mBio.00145-21)

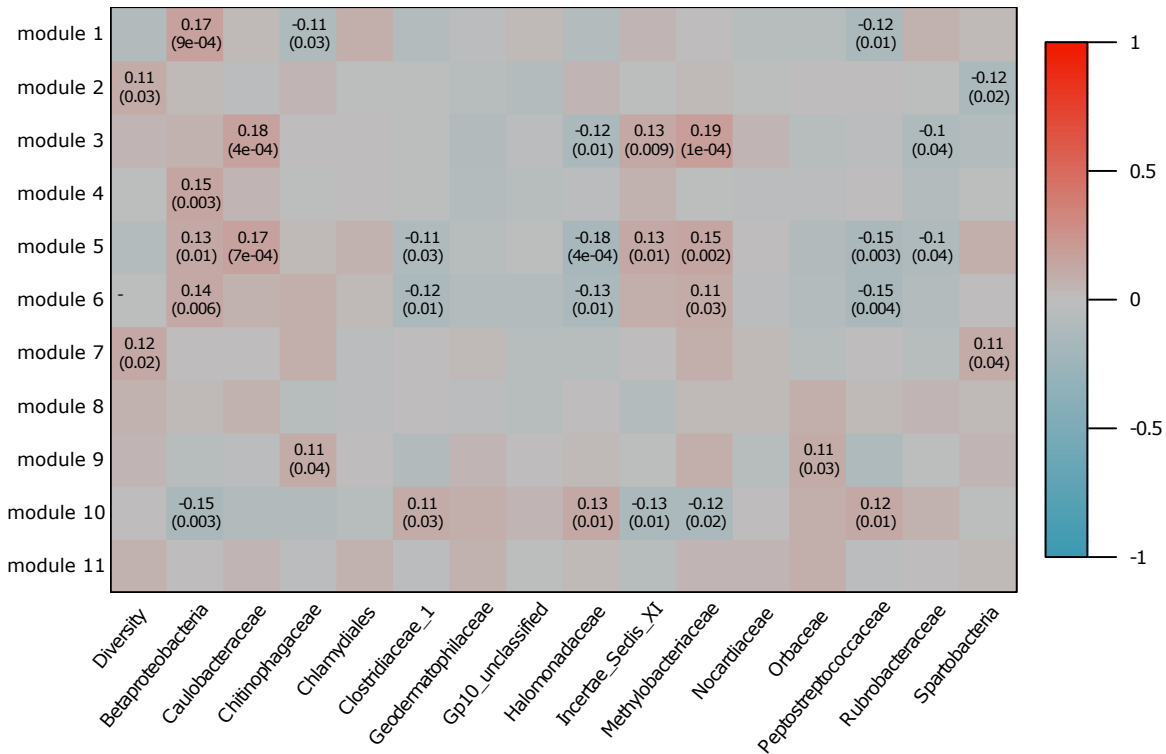

Supplement: FIG S2 [file mbio.00145-21-sf002.pdf]

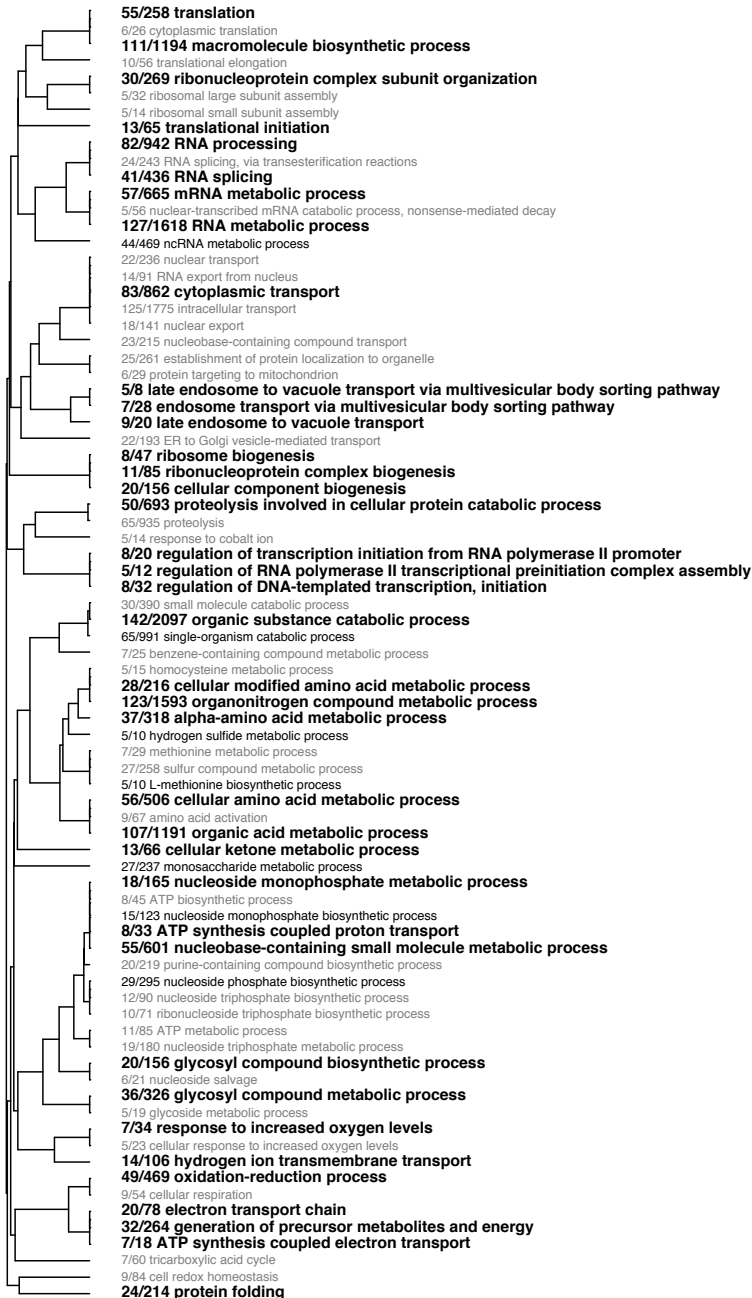

p < 0.005  
p < 0.01  
p < 0.05

Supplement: FIG S3 [file mbio.00145-21-sf003.pdf]

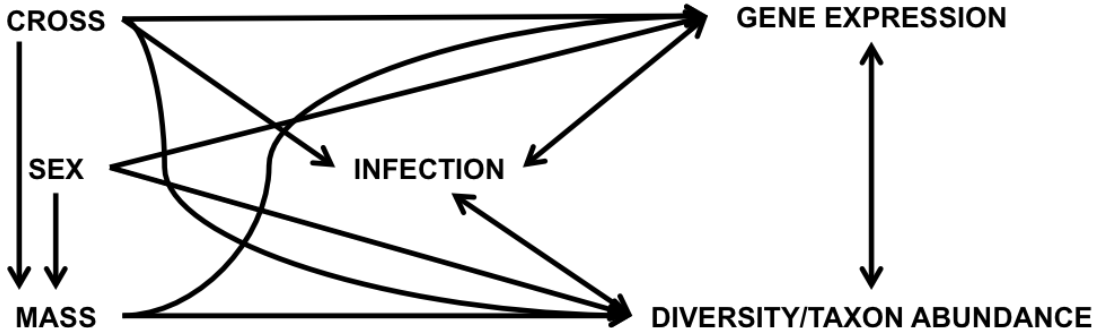

Supplement: FIG S1 [file mbio.00145-21-sf001.pdf]
